# VapC from the Leptospiral VapBC Toxin-Antitoxin Module Displays Ribonuclease Activity on the Initiator tRNA

**DOI:** 10.1371/journal.pone.0101678

**Published:** 2014-07-21

**Authors:** Alexandre P. Y. Lopes, Luana M. Lopes, Tatiana R. Fraga, Rosa M. Chura-Chambi, André L. Sanson, Elisabeth Cheng, Erika Nakajima, Ligia Morganti, Elizabeth A. L. Martins

**Affiliations:** 1 Centro de Biotecnologia, Instituto Butantan, São Paulo, São Paulo, Brazil; 2 Centro de Biotecnologia, Instituto de Pesquisas Energéticas e Nucleares, Comissão Nacional de Energia Nuclear, São Paulo, São Paulo, Brazil; University of Strathclyde, United Kingdom

## Abstract

The prokaryotic ubiquitous Toxin-Antitoxin (TA) operons encode a stable toxin and an unstable antitoxin. The most accepted hypothesis of the physiological function of the TA system is the reversible cessation of cellular growth under stress conditions. The major TA family, VapBC is present in the spirochaete *Leptospira interrogans*. VapBC modules are classified based on the presence of a predicted ribonucleasic PIN domain in the VapC toxin. The expression of the leptospiral VapC in *E. coli* promotes a strong bacterial growth arrestment, making it difficult to express the recombinant protein. Nevertheless, we showed that long term induction of expression in *E. coli* enabled the recovery of VapC in inclusion bodies. The recombinant protein was successfully refolded by high hydrostatic pressure, providing a new method to obtain the toxin in a soluble and active form. The structural integrity of the recombinant VapB and VapC proteins was assessed by circular dichroism spectroscopy. Physical interaction between the VapC toxin and the VapB antitoxin was demonstrated *in vivo* and *in vitro* by pull down and ligand affinity blotting assays, respectively, thereby indicating the ultimate mechanism by which the activity of the toxin is regulated in bacteria. The predicted model of the leptospiral VapC structure closely matches the *Shigella*'s VapC X-ray structure. In agreement, the ribonuclease activity of the leptospiral VapC was similar to the activity described for *Shigella*'s VapC, as demonstrated by the cleavage of tRNA^fMet^ and by the absence of unspecific activity towards *E. coli* rRNA. This finding suggests that the cleavage of the initiator transfer RNA may represent a common mechanism to a larger group of bacteria and potentially configures a mechanism of post-transcriptional regulation leading to the inhibition of global translation.

## Introduction

Toxin-Antitoxin (TA) systems consist of operons coding for an unstable antitoxin and a stable toxin. The toxin is blocked by the antitoxin, unless some environmental condition determines a decrease in antitoxin concentration, resulting in exposure of the cell to the toxic effects [1]–[3].

Three different types of TA modules are described: I- the antitoxin is an antisense RNA to the mRNA coding the toxin, inhibiting its translation [4], [5]; II- toxin and antitoxin interact at protein level; and III- the antitoxin is an RNA which binds directly to the toxic protein [6].

Overexpression of toxins can cause inhibition of cellular growth and death by targeting key molecules in several essential processes, including DNA replication [7], mRNA stability [8], selective or general protein synthesis [9], cell wall and ATP synthesis [10], cytoskeleton proteins polymerization and cell division [11].

The physiologic function of these TA modules, more than promoting a programmed cell death, has been consensually related to stress management [12], [13], inducing protective dormancy (reversible cessation of proliferation), biofilm formation and multidrug tolerance – the persisters [14]–[16].

The type II TA modules are the most abundant and have been grouped in 14 different families according to the toxin structure and protein sequence similarity [17]. VapBC (virulence associated proteins B and C) is the major TA type II family (about 1,900 VapBC modules were identified in ∼960 genomes), counting 30 to 40% of known TAs (URL: http://bioinfo-mml.sjtu.edu.cn/TADB/) [17], [18]. They are classified based on the presence of a PIN (PilT N-terminal) domain in VapC, which is predicted to have ribonuclease activity [19]. VapCs, like the toxins of the families RelBE, MazEF and HicAB, has been described as endoribonucleases, also called RNA interferases [20], [21].

Several studies have confirmed the RNAse activity of VapCs towards synthetic or total RNA extracts [8], [22]–[24]. However, the specific targets of these toxins and their precise mechanisms of action remain mostly unknown. Recently, it was reported that VapCs from the enteric bacteria *Shigella flexneri* and *Salmonella enterica* cleave specifically the anticodon stem loop of the initiator N-formyl-methionyl-tRNA (tRNA^fMet^) in a single bond between nucleotides A38 and C39 [25], and that VapC20 from *Mycobacterium tuberculosis* cleaves Sarcin-Ricin loop of 23S rRNA between nucleotides G2661 and A2662 [26]. Messenger RNAs controlling specific physiological functions were also shown to be possible VapC targets in *Sulfolobus solfataricus* and *Mycobacterium tuberculosis* [27], [28].

Although conservation within VapCs primary structures is considered to be poor, the PIN domain structural fold is conserved. The PIN domain is described as a 3-layer α/β/α sandwich containing 5-stranded β-sheet in the center of the structure that brings together a cluster of 3 or 4 acidic residues with an invariant serine or threonine residue responsible for coordinating Mg^+2^ or Mn^+2^ ions in the catalytic site [19], [29].

The analysis of the genomes of pathogenic and saprophyte *Leptospira* strains [30]–[32] enabled the identification of proteins from different TA families: *mazEF*; *chpKI* [33], [34]; and *vapBC* [35]. In *Leptospira interrogans* serovar Copenhageni strain Fiocruz L1-130, one of the causative agents of human leptospirosis [36], [37], four VapBC modules were identified by TADB integrated database (URL: http://bioinfo-mml.sjtu.edu.cn/TADB/).

Due to the toxicity of VapC over the bacterial host, biochemical studies of the toxin have been frequently performed using either the recombinant toxin-antitoxin complex (VapB-VapC) [8], [22] or the complex after trypsin hydrolysis of VapB [23], [38] or yet by denaturing the complex immobilized through His-tagged VapB, followed by refolding of VapC [25].

In this work we present a new strategy to obtain functional and active VapC consisting of long term expression of the insoluble protein in inclusion bodies, followed by solubilization and refolding by high hydrostatic pressure (HPP). We have also provided experimental evidence of the physical interaction between VapB and VapC from *Leptospira interrogans*. Moreover, we have shown through functional and structural parameters that the leptospiral VapC is closely related to the enteric VapCs sharing the same molecular target; thus, contributing to consolidate the knowledge on the features of the TA modules.

## Materials and Methods

### Bacterial strains and culture conditions

*E. coli* DH5α and *E. coli* BL21(DE3)Star[pLysS] (Novagen) were used for gene cloning and protein expression, respectively. *E. coli* strains BL21(DE3)C43 and BL21(DE3)trxB were also tested for expression of VapC. The clones were grown in 2YT medium with 100 µg.ml^−1^ of ampicillin (2YT+Amp).

### Cloning the genes *vapB* and *vapC*

The genes *vapB* (locus tag LIC12659; Gene ID 2769629), *vapC* (locus tag LIC12660; Gene ID 2769615) or both *vapBC* were amplified by PCR from genomic DNA of *Leptospira interrogans* serovar Copenhageni strain Fiocruz L1-130 [31], using the primers listed in Table 1. PCR products were cloned in pGEM-T Easy plasmid (Promega) and subcloned in the expression vector pAE [39], which allows expression of 6×His-tagged proteins (Table 1). Constructions were confirmed by DNA sequencing (ABI Prism 3100×L sequencer Seq-Wright) using primers T7 forward (5′ TAATACGACTCACTATAGGG 3′) and pRSET reverse (5′ ATGCTAGTTATTGCTCAGCGGTGG 3′).

**Table 1:** Primers used in the amplification of leptospiral genes and expression plasmids.

Genes	Primers *	Restriction enzymes*	Plasmids
LIC12659 (*vapB*)	F: 5′GGATCCATGCAAACAGCCAAATTATTTA3′	BamHI	pAE-*vapB*
	R: 5′AAGCTTTAAGATTTTCTAACAGAGTCGC3′	HindIII	
LIC12660 (*vapC*)	F: 5′CTCGAGTTAATGTATCTTTTGGATACC3′	XhoI	pAE-*vapC*
	R: 5′GACGTCTTACTACTGTGTCCAATTTTC3′	PstI	
LIC12659-LIC12660 (*vapBC*)	F: 5′GGATCCATGCAAACAGCCAAATTATTTA3′	BamHI	pAE-*vapBC*
	R: 5′GACGTCTTACTACTGTGTCCAATTTTC3′	PstI	

* The restriction sites for the enzymes are underlined in the primers.

### Expression of the recombinant proteins and *E. coli* growth kinetic

Clones of *E. coli* BL21 cells transformed with pAE, pAE-*vapB*, pAE-*vapC* or pAE-*vapBC* were cultured in 500 ml of 2YT+Amp medium until optical density at 600 nm reached 0.6. Protein expression was induced by IPTG (1 mM) and growth was continued for 4 h at 37°C for VapB and VapBC; and for 16 h at 30°C for VapC. The cells were separated by centrifugation (2,500×*g* for 10 min at 4°C), suspended in lysis buffer (50 mM Tris-HCl pH 8.0, 150 mM NaCl, 5 mM imidazole) and disrupted by sonication or French Press. Soluble and insoluble fractions of the extracts were separated by centrifugation (8,500×*g* for 10 min at 4°C) and samples were analyzed by SDS-PAGE. The relative amount of soluble and insoluble recombinant proteins was estimated by the relative intensity of the bands in the polyacrylamide gels, measured by densitometry (BioRad GS-800 densitometer - Quantity One 4.6.3 software - Biorad Life Sciencs, USA) and considering the volume of soluble and insoluble fractions in relation to the volume of the total extract. The kinetic of *E. coli* growth was followed by collecting culture samples every 30 minutes and measuring the optical density at 600 nm. *E. coli* carrying empty pAE vector was used as negative control.

### Refolding of VapC using high hydrostatic pressure (HHP)

Refolding of VapC was performed as previously described with few modifications [40].

#### Isolation and preparation of VapC inclusion bodies for pressurization assays

After induction, bacterial cells from 500 ml *E. coli* culture were collected by centrifugation and resuspended in 50 ml of lysis buffer containing 50 µg/ml lysozyme. After 15 min of incubation at room temperature (RT) the cells were disrupted by French Press (Thermo Spectronic) and suspension was centrifuged at 8,500 g for 10 min at 4°C. The insoluble fraction (inclusion bodies – IBs) was washed sequentially with 30 ml of Buffer A (100 mM Tris-HCl, pH 8.0, 5 mM EDTA, containing 0.1% [w/v] sodium deoxycholate), Buffer B (50 mM Tris-HCl, pH 8.0, 1 mM EDTA, 100 mM NaCl, 0.25 M urea) and Buffer C (50 mM Tris-HCl, pH 8.0, 1 mM EDTA). The pellet was resuspended in 10 ml of Buffer C (basic refolding buffer) and stored at −20°C for further assays.

#### Formulation of refolding buffers and VapC pressurization assays

Samples of the IBs suspension were diluted in refolding buffer containing different concentrations of L-arginine (0.25, 0.5 and 1 M), guanidine hydrochloride (0.1, 0.25 and 0.5 M), and oxidized (GSSG) and reduced (GSH) glutathiones (1∶1; 250 mM final concentration). One ml samples of the suspensions were placed into plastic bags which were sealed and placed in other plastic bags that were then vacuum/heat-sealed. The bags were positioned in a pressure vessel (model R4-6-40, High Pressure Equipment Company) with a mixture of water and oil as a pressure-transmitting fluid and high pressure (200 MPa, 2000 bar, or 29,000 psi) was applied for 16 h. After pressure release, the samples were centrifuged at 12,000 g for 15 min. The supernatants were dialyzed against 50 mM Tris-HCl buffer, pH 8.0 and centrifuged (12,000 g for 15 min) to remove insoluble aggregates eventually formed. The buffer containing 0.5 M L-arginine, which resulted in higher yield, was selected for refolding of VapC IBs by pressurization.

### Purification of recombinant proteins by immobilized metal ion affinity chromatography (IMAC)

The soluble fraction of *E. coli* extracts expressing VapB (pAE-*vapB*), VapB and VapC (pAE-*vapBC*) or the solution containing the pressurized VapC (pAE-*vapC*) were applied to a 1 ml Ni^+2^-Sepharose Histrap HP column (GE Healthcare) previously equilibrated with 50 mM Tris-HCl pH 8.0, 150 mM NaCl, 5 mM imidazole. Proteins were eluted using 50 mM Tris-HCl pH 8.0, 150 mM NaCl, 250 mM imidazole and fractions were analyzed by SDS–PAGE, pooled and dialyzed against 50 mM Tris-HCl, pH 8.0. Total protein concentration was determined by Bradford assay.

### Circular dichroism (CD) spectroscopy

Samples of 10 µM of protein solution in 20 mM Na-phosphate buffer, pH 7.4 were submitted to CD measurements using a J-810 Circular Dichroism Spectropolarimeter (Jasco Inc.). The data represent the average over five scans of far-UV spectra (195–255 nm) collected at 20°C. Spectral corrections were performed subtracting the blank. The measure of ellipticity *θ* (mdegree) was converted to molar mean residue ellipticity, [*θ*] (degree.cm^2^.dmol^−1^). Predictions of the secondary structure were performed by the program PSIPRED (URL: http://bioinf.cs.ucl.ac.uk/psipred/).

### Ethics statement

This work used mice for obtaining VapB anti-serum. During the experiment, animals were supplied with food and water *ad libitum* and experimental protocols were previously approved by the Ethical Committee for Animal Research of the Butantan Institute, under the license number 252/06.

### Production of VapB anti-serum and IgG purification

BALB/c mice were provided by the animal facility from Instituto Butantan. BALB/c female mice were immunized by three intraperitoneal injections of 5 µg of VapB, containing the adjuvant aluminium hidroxide, with a two week interval between each other. Fifteen days after the last injection, mice were bled from the retro-orbital plexus and serum was separated by centrifugation (2,300×*g* for 3 min at 4°C). IgG fraction was purified using Protein A-Sepharose according to manufacturer's instruction (GE Healthcare). Negative control serum was prepared in the same way from blood of animals injected with PBS and adjuvant only.

### Ligand affinity blotting assay

Binding of VapC with VapB was assessed by ligand affinity blotting [41], [42]. One µg of VapC was subjected to 15% SDS-PAGE and transferred to nitrocellulose membrane, which was blocked for 16 h with 10% non-fat milk in PBS-T (PBS pH 7.4 containing 0.05% Tween 20). The membrane was exposed to a VapB solution (3 µg ml^−1^ in PBS) for 1 h. After extensive washing with PBS-T, the membrane was incubated with purified anti-VapB antibodies (diluted 1∶100) for 1 h. The membrane was washed with PBS-T and incubated with anti-mouse IgG conjugated with horseradish peroxidase (KPL) (diluted 1∶5,000) for 1 h. After washing, bound antibodies were detected using Super Signal West Pico Chemiluminescent Substrate (Pierce). The leptospiral protein LipL32 was used as negative control. Similar ligand affinity blotting was performed using negative control serum. In addition, a Western blot was performed to verify the recognition of VapB and not VapC or LipL32 by the anti-VapB antibodies.

### Ribonuclease (RNase) activity of VapC

The ribonuclease activity of the refolded VapC was tested towards *E. coli* rRNA or tRNA^fMet^ as described [8], [25] with few modifications. *rRNA assay*: total RNA was extracted from an *E. coli* culture (OD_600nm_ 0.8) using Trizol reagent (Invitrogen) according to the manufacturer's instructions. The purity and concentration of the preparation containing mostly ribosomal RNA (rRNA) was measured by spectrophotometry at 260 and 280 nm. Aliquots of purified VapC (2.5 and 5 pmol) were incubated with 1 µg of rRNA in reaction buffer (10 mM Hepes pH 7.5, 15 mM KCl, 1 mM DTT and 10% glycerol) at 37°C for 10 min in the presence or absence of 1 mM Mg^+2^ or Mn^+2^, in 10 µl final volume. The reactions were stopped by adding 2 µl 50% formamide and analyzed by electrophoresis in 1% Tris-Acetic-EDTA agarose gel with 1 µg/ml ethidium bromide. *tRNA^fMet^ assay*: the initiator *E. coli* tRNA^fMet^ was acquired from Sigma-Aldrich. VapC (1.25 to 5 pmol) was incubated with 3 pmol of tRNA^fMet^ in the reaction buffer at 37°C for 30 min. VapC activity was tested in presence of either EDTA or ions Mg^+2^ or Mn^+2^ (0.01 to 100 mM). Inhibition of VapC was tested by pre-incubating it with VapB (0.8 to 6.4 pmol). The reactions were stopped using 2 µl 50% formamide and analyzed by denaturing 8% PAGE 6 M urea, stained with ethidium bromide.

### Three-dimensional (3D) modeling

Sequence alignments were performed using the blast tool of NCBI at http://blast.ncbi.nlm.nih.gov/Blast.cgi. VapC 3D structures were constructed using the “alignment mode” of SWISS-MODEL automated protein structure homology-modelling server (http://swissmodel.expasy.org) [43]. The target amino acid sequences were submitted to a modeling process based on a user-defined target-template alignment. The model was handled with the program “DeepView” - project mode - (Swiss-PdbViewer), an integrated sequence-to-structure workbench. The quality of modelling was evaluated in the SwissModel Workspace by QMEAN4 (Qualitative Model Energy Analysis) and Z score [44].

## Results

### The *vapBC* operon from *Leptospira interrogans* serovar Copenhageni

The *vapB* and *vapC* genes studied in this work are localized *in tandem* in the Chromosome I of the pathogenic *L. interrogans* serovar Copenhageni strain Fiocruz L1-130 [31]. The initial characterization of a leptospiral VapBC module was made by Zhang et al. [35] with genes from *L. interrogans* serovar Lai, which differs by a single amino acid in VapB (E64G) from the homolog studied here. These authors showed, through phylogenetic comparison among different bacteria and archaea, that lateral transfer of TA genes have occurred, confirming straight co-evolution of VapB and VapC. As shown below, cloning leptospiral *vapBC* locus under control of a single promoter in pAE vector in *E. coli* allowed the expression of both proteins simultaneously, indicating that the bicistronic mRNA was processed the same way it is processed in *Leptospira*.

### Soluble and available VapC influences the growth rate of *E. coli*

**Figure 1:**
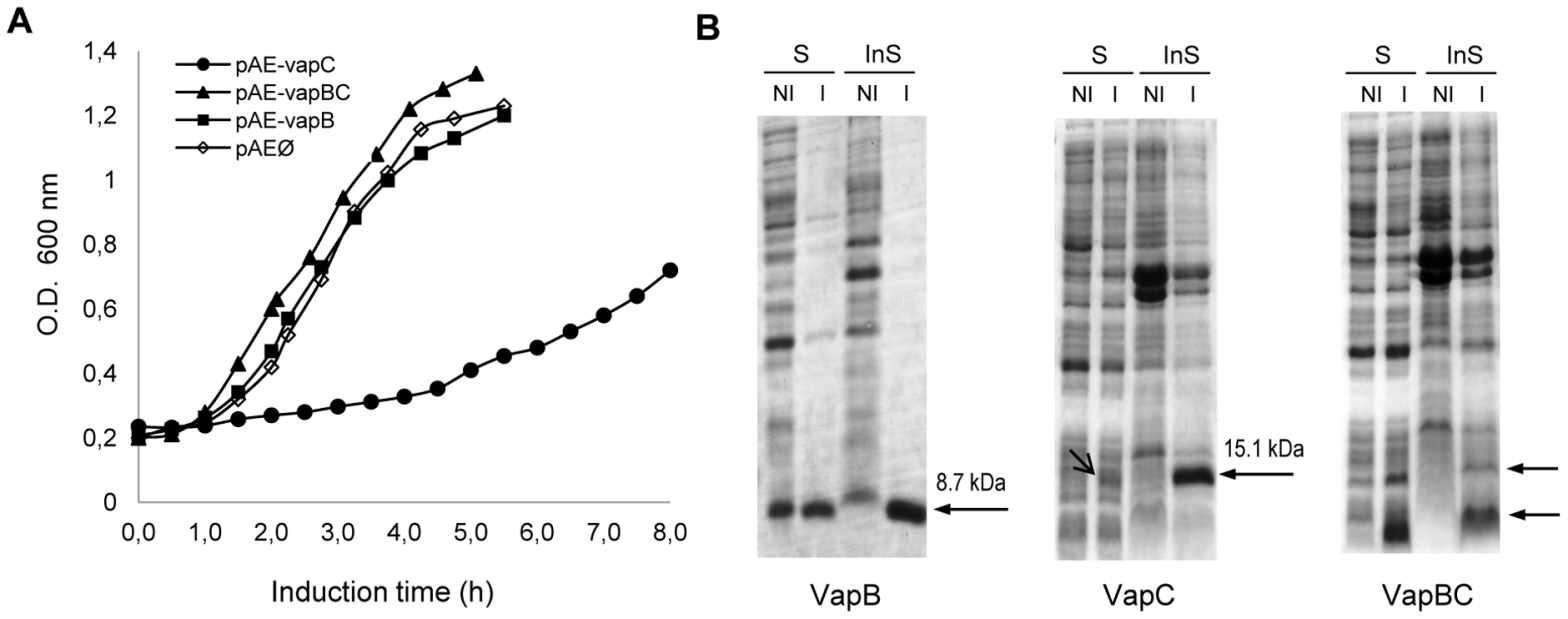
Solubility and availability of VapC influences the growth rate of *E. coli*. (**A**) The growth of *E. coli* transformed with control pAEØ (◊), pAE-*vapB* (▪), pAE-*vapC* (•) or pAE-*vapBC* (▴) was followed after induction, for 5 h to 8 h. *E. coli* harboring the empty vector pAE was used as control. The data represents one experiment that was reproduced at least three times in the laboratory. (**B**) Samples (10 µl) were applied to SDS-PAGE to analyze the expression of soluble [S] or insoluble [InS] proteins from induced [I] and not induced [NI] cultures. The arrows indicate the recombinant proteins. The relative amount of soluble and insoluble VapB and VapC proteins were estimated by densitometry of the bands in the gel, considering that the insoluble fraction was resuspended 5× concentrated in relation to the soluble fraction. VapB was expressed predominantly (∼80%) in soluble form (▪). *E. coli* growth was arrested by the expression of VapC (•), probably due to the toxic effect of the protein in soluble and active form (∼10%). Co-expression of the VapB antitoxin (▴) restored the bacterial growth and increased the expression of the soluble form of VapC (∼89%).

Clones of *E. coli* transformed with the plasmids pAE, pAE-*vapB*, pAE-*vapC* or pAE-*vapBC* were cultured and recombinant protein expression was induced with IPTG. As reported for VapC from *L. interrogans* serovar Lai [35], a strong growth arrestment of *E. coli* occurred when the VapC toxin was expressed solely (Fig. 1A - •), and it was normalized by the co-expression of VapB (Fig. 1A - ▴).The inhibition of the growth of *E. coli* carrying heterologous *vapC* was described in several other studies [8], [35], [45], and as observed in our experiments, a slow and gradual recovery of cellular growth rate occurs with longer incubation times (Fig. 1A). We hypothesized that the cells have overcome the toxic effects of VapC by aggregating the toxic protein in inclusion bodies. In fact, almost the totality of VapC from *E. coli* culture was found as insoluble protein, but when VapB is co-expressed, its solubility rises significantly without exerting the expected toxicity (Fig. 1B), once the availability of the toxin was compromised due to its interaction with the antitoxin, as shown below.

### Production of the recombinant VapB and VapC proteins

The VapB antitoxin and the VapC toxin, expressed separately, were mostly recovered in the soluble and in the insoluble fractions of *E. coli* extracts, respectively (Fig 1B).

#### VapC was successfully refolded by high hydrostatic pressure

In order to obtain soluble VapC, several conditions of bacterial culture and protein expression were tested, such as temperature variation (20°C, 30°C and 37°C), time of induction (1 h, 5 h and 14 h), and IPTG concentrations (0.5 mM and 1 mM). Different strains of *E. coli* were also tested as host (BL21(DE3)C43, BL21(DE3) trxB and BL21(DE3)Star[pLysS]), without success. Our attempts to refold VapC by the usual methodology of solubilization at full denaturing condition with urea and dialysis after metal affinity chromatography purification also failed. In fact, due to the toxicity of VapC for the host bacteria, its expression and isolation in soluble form have been found not suitable, unless it was co-expressed with the cognate VapB antitoxin [8], [19], [22], [23].

As described above, *E. coli* can gradually overcome VapC toxicity and, therefore, to increase bacterial density, the induction was kept during longer time (∼16 h), thereby enabling the recovery of the protein. To obtain soluble and biologically active VapC from the inclusion bodies, a refolding step using HHP (200 MPa) was applied. The use of HHP was previously described as an interesting alternative for the traditional refolding processes at atmospheric pressure [46], [47]. The thermodynamic mechanism by which high pressure enhances protein refolding has been extensively studied, including the effects of chaotropes and temperature. Protein aggregates are frequently less dense than native proteins. High pressure modulates protein thermodynamics by favoring the decrease of the system volume, the dissociation of aggregates and subsequent formation of native, more compact structures. Mechanistically, high pressure treatment is thought to dissociate aggregates by insertion of water molecules within hydrophobic protein-protein interfaces, leading to disaggregation and subsequent refolding [48], [49]. The method allows refolding of proteins with high yields [50], [51] likely because the conditions of protein solubilization preserve the native-like secondary and tertiary structures that exist in proteins in the inclusion bodies, favoring the complete refolding [52].

**Figure 2:**
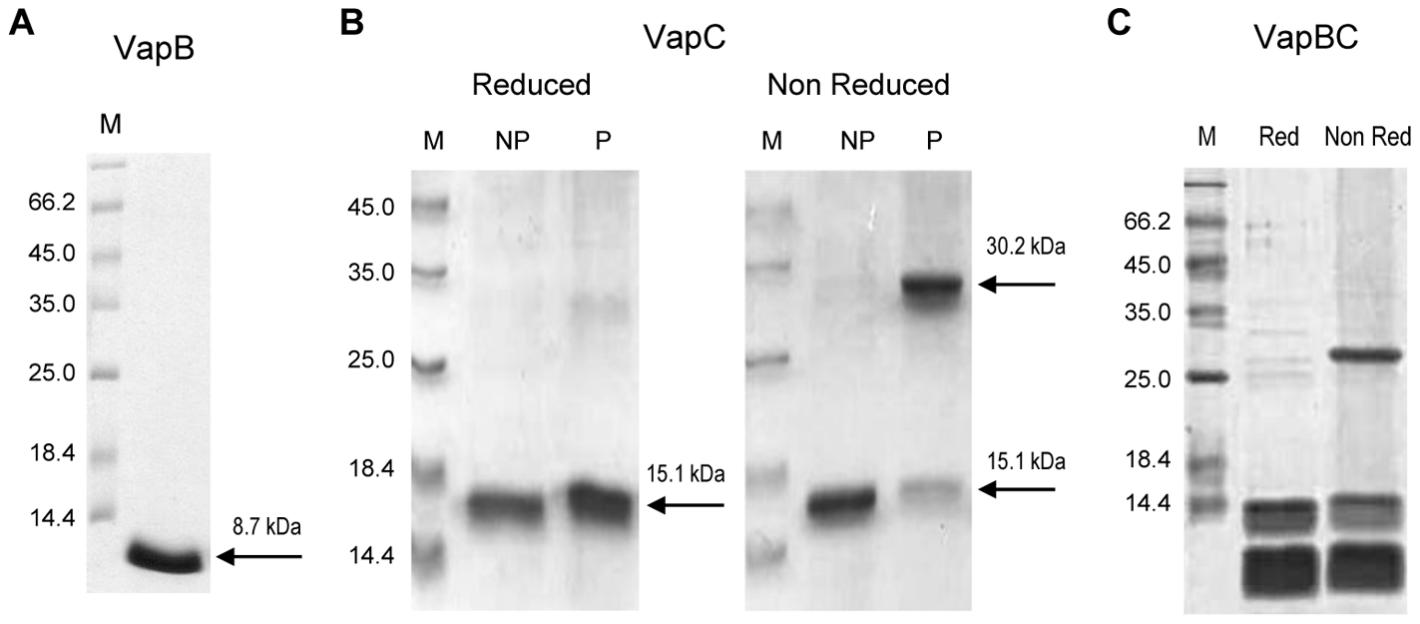
Analysis of purified recombinant proteins by SDS-PAGE revealed VapC dimerization. (**A**) VapB purified from the soluble fraction of *E. coli* extracts (**B**) VapC expressed as inclusion bodies, before [NP] or after [P] pressurization in buffer containing 0.5 M L-arginine; (**C**) VapB and VapC purified from the soluble fraction of *E. coli* co-expressing both proteins. Samples were prepared with or without β-mercaptoethanol ([Reduced] or [NReduced], respectively). M - Molecular Marker (kDa). The arrows indicate purified VapB and monomeric (15.1 kDa) or dimeric (30.2 kDa) forms of VapC. Remarkably, VapC dimers were observed in non-reduced samples of the pressurized protein (B) and in the co-purification with VapB (C). This analysis was made with 3 VapC preparations.

VapC inclusion bodies suspension was pressurized in solutions containing different concentrations of guanidine hydrochloride or L-arginine, with and without redox shuffling agents (reduced and oxidized glutathione). The solubilization and refolding of VapC were achieved by pressurization in presence of L-arginine, which is a widely used additive in protein refolding. It can bind to partially folded or denatured proteins, inhibiting intermolecular hydrophobic interactions, thus acting as a chaperone [40], [53]. The refolding of VapC yielded 98% as estimated by densitometry of the bands in the SDS-PAGE (Fig. 2B). The VapC solubilized under this condition was used for the further investigations.

#### Recombinant leptospiral VapC dimerizes through intermolecular disulfide bond

Soluble fraction of *E. coli* extracts expressing VapB or refolded VapC obtained by HHP were purified by metal affinity chromatography and analyzed by SDS-PAGE (Fig. 2A and 2B). In agreement with the predicted molecular weight, reduced VapC migrates as a 15.1 kDa molecule, while under non-reducing conditions a new band appears to present twice this molecular weight (Fig. 2B), indicating that the only cystein residue (Cys9) of VapC molecule is able to form intermolecular disulfide bonds, therefore producing dimers.

In order to check whether VapC dimerization would also happen *in vivo* during ectopic expression, samples of the proteins purified from extracts of *E. coli* co-expressing VapB and VapC we analyzed by SDS-PAGE and it was verified that the same dimerization phenomenon occurred (Fig. 2C). It shall be remarked that the presence of dimers was observed in overexpression conditions and therefore one cannot assume or exclude that it is a natural phenomenon in *L. interrogans*. Although it has been reported that VapC, as most of the PIN domain proteins, appears as dimers in solution [54], intermolecular disulfide bonds has never been shown. Interestingly, we found that the ribonuclease activity of VapC increases in the presence of the reducing agent DTT (data not shown). Taking these data together, we consider that the formation of such disulfide-bound dimers might be an artifact or even some kind of regulation based on redox potential in cells.

#### The recombinant proteins are correctly folded

**Figure 3:**
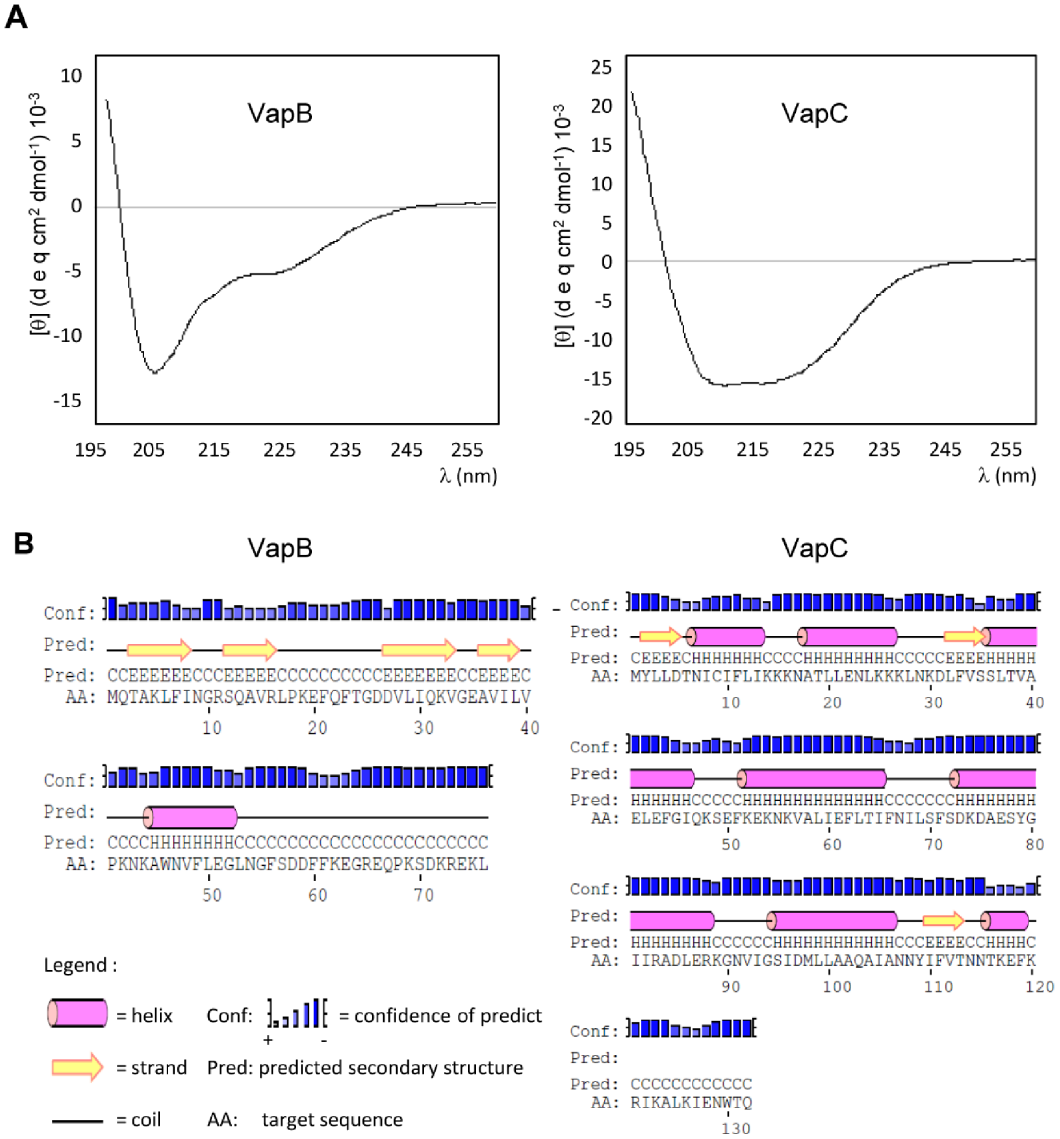
Circular dichroism of VapB and VapC confirmed predicted secondary structure. (**A**) The VapB and VapC CD spectra were recorded in the wavelength range of 195–255 nm as average of five scans at 20°C. Measured ellipticities, *θ* (mdegree), were converted to molar mean residue ellipticities, [*θ*] (degree.cm^2^.dmol^−1^). The assays were reproduced with at least 2 samples of each protein. (**B**) Prediction of secondary structure by the PSIPRED algorithm using the primary sequence of the proteins. The experimental data confirmed the secondary structure predicted by computational analysis.

The structure analysis of VapB and VapC by far-UV CD spectroscopy indicated that the proteins are likely to be folded. A mixture of α-helix, β-strand and coil regions in VapB structure and a predominance of α-helices in VapC was denoted by the minima ellipticities at 208 and 222 nm in the dichroism spectrum (Fig. 3A). The experimental data on the secondary structure is in agreement with the prediction of computational analysis (PSIPRED) (Fig. 3B).

### The recombinant VapB and VapC proteins interact *in vivo* and *in vitro*

**Figure 4:**
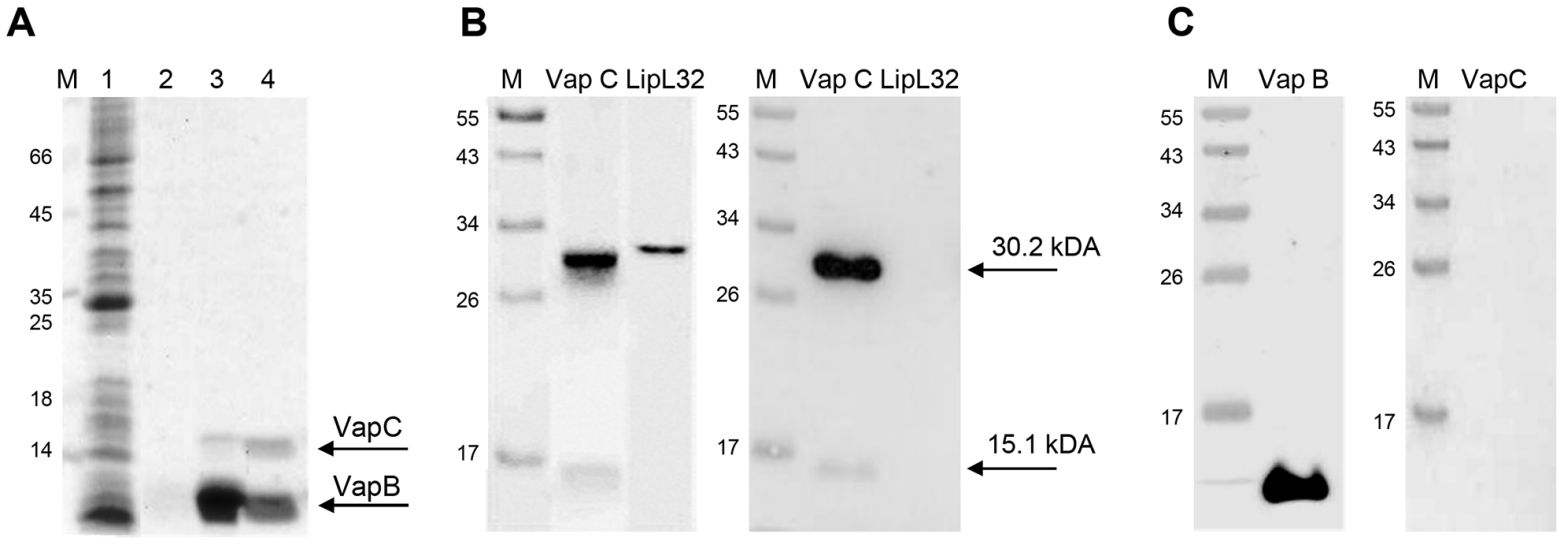
VapB and VapC interact *in vivo* and *in vitro*. (**A**) Pull-down assay. The soluble fraction of *E. coli* pAE*vapBC* extract was applied to a Ni^+2^-Sepharose column. Samples were analyzed by SDS-PAGE. Lane 1: initial sample; lane 2: washing; lanes 3–4: elution with 250 mM imidazole. It is important to observe that no VapC was released during the washing step, being co-purified with VapB-His, denoting the *in vivo* interaction. The arrows indicate the VapB and VapC bands. M - Molecular Weight Marker (kDa). Pull-down assay was perfomed more than 5 times. (**B**) Ligand affinity blotting. To analyze specific binding between VapB and VapC, the VapC and LipL32 proteins (negative control) were subjected to 15% SDS-PAGE (left panel) and transferred to nitrocellulose membrane (right panel). After blocking, the membrane was incubated with a VapB solution (3 µg ml^−1^). Following extensive washing, the membrane was incubated with anti-VapB antibodies. M - Prestained Molecular Weight Marker (kDa). VapB in solution bound to both monomeric (15.1 kDa) and dimeric (30.2 kDa) forms of VapC immobilized in the membrane, denoting *in vitro* interaction. VapB did not bound the negative control, protein LipL32.(**C**) Western blot control showing that anti-VapB antibodies recognizes specifically VapB, and not VapC.

To verify whether there is a physical interaction between recombinant VapB and VapC proteins during ectopic expression *in vivo*, a pull-down assay was performed. The soluble fraction of induced *E. coli*-pAE-*vapBC* extracts was applied to a Ni^+2^-Sepharose column. SDS-PAGE analysis of eluted fractions revealed that VapB and VapC were co-purified (Fig. 4A), meaning that the His-tagged VapB immobilized by metal affinity was able to bind non-tagged VapC in solution and, as result of their affinity, both proteins were pulled-down from the column with high imidazole concentrations. This result indicates that the interaction between these proteins may be responsible for the observed neutralization of VapC toxicity (Fig. 1A), when co-expressed with VapB in *E. coli*.

The *in vitro* interaction of purified VapB and refolded VapC was evaluated by ligand affinity blotting assay [41], [42]. VapC was subjected to SDS-PAGE and transferred to a nitrocellulose membrane, which was exposed to a solution containing VapB. The VapB retained in the membrane was detected by anti-VapB specific antibodies, indicating that it was able to bind both monomeric and dimeric forms of immobilized VapC (Fig. 4B). Notably, VapB did not bind to LipL32, used as negative control, and anti-VapB antibodies did not recognize VapC directly (Fig. 4C), supporting our conclusions that VapB interacts specifically with VapC.

### The 3D model of leptospiral VapC matches closely with experimental X-ray structure of *Shigella*'s VapC

Considering that the 3D structure of an enzyme correlates to its biochemical activity, we have submitted the primary sequence of VapC from *L. interrogans* to 3D modelling (SWISS MODEL) using the experimentally solved X-ray structures of VapCs as templates: *Shigella flexneri* VapC - PdB: 3TND/C; *Neisseria gonorrhoeae* FitAB – PdB: 2H1C/A [55]; *Mycobacterium tuberculosis* VapC5 – PdB: 3DBO [22], [56]; *M. tuberculosis* VapC3 – PdB: 3H87/B [56]; and *Pyrobaculum aerophilum* PAE0151 – PdB: 2FE1A. Additionally, the models of VapC from *Salmonella enterica* and VapC20 from *M. tuberculosis* were analyzed.

To compare and rank alternative models of the same target protein, the models created were evaluated by QMEAN4 and Z scores. QMEAN4 is a reliability score composed of a linear combination of four structural descriptors using statistical potentials: the local geometry is analyzed by a torsion angle potential over three consecutive amino acids; two distance-dependent interaction potentials are used to assess long-range interactions; and a solvation potential investigates the burial status of the residues. Z-score represents a measure of the absolute quality of a model providing an estimate of the “degree of nativeness” of the structural features observed and indicates whether the model is of comparable quality to experimental structures. [44]. QMEAN4 estimates quality ranges between 0 and 1 with higher values for better models. Higher *Z*-scores consistently relate to favorable states reaching for “good quality” models a mean *Z*-score  = −0.65, for “medium quality” models a mean *Z*-score  = −1.75 and for “low-quality” models a mean *Z*-score  = −3.85 [44]. It should be mentioned that the crystal of *Shigella*'s VapBC which was one of the templates used in this analysis, revealed a hetero-octameric assembly (VapB_4_C_4_) [57] and that slight differences in each monomer are observed among the structures of each subunit, consequently the scores produced can vary slightly according to the chosen chain; the same occurring to other multimeric structures.

**Figure 5:**
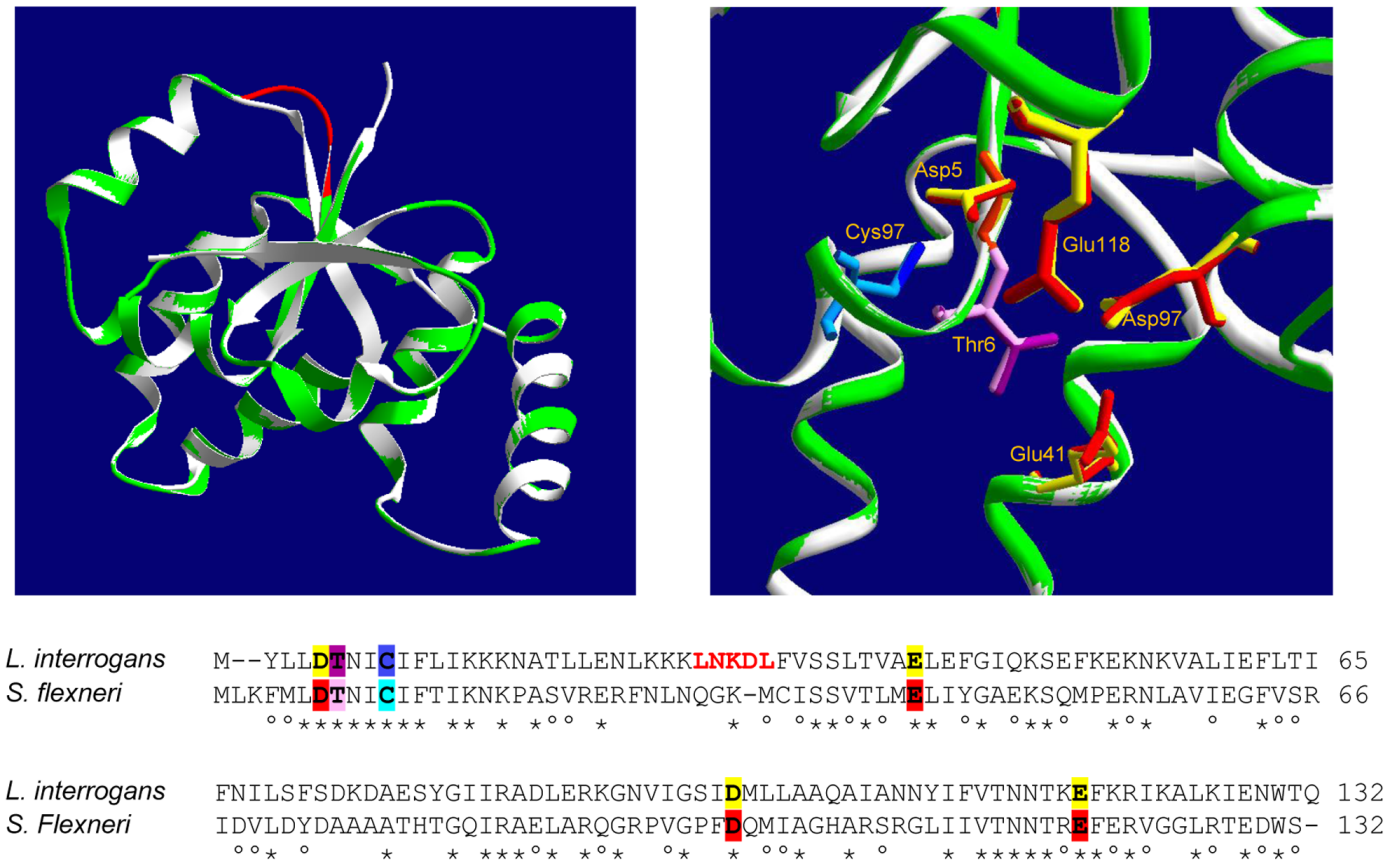
3D model of leptospiral VapC closely matches the experimental X-ray structure of *Shigella*'s VapC. Alignment of the sequences of VapCs from *S. flexneri* and *L. interrogans* is shown. On the left panel, the structure model (ribbon) of VapC from *L. interrogans* (green) was superimposed to the VapC template from *S. flexneri* (white) (PdB: 3TND-C). The green regions in the target appear almost identical to the template, while the red region does not correspond. The amino acids composing the red region are written in the same color in the sequence alignment. On the right panel, superimposition shows the perfect matching of the conserved threonine and the four acidic residues responsible for coordinating metal ions in the catalytic site, and the cysteine involved in dimerization, positioned in the neighborhood of the catalytic site. These six residues are numbered in the structure according to leptospiral VapC sequence and colored as highlighted in the alignment.

The *in silico* 3D structural model of leptospiral VapC matched quite perfectly with the template structure of *Shigella*'s VapC, as revealed by the superimposition of the structures (Fig. 5). The conserved residues responsible for coordinating metal ions in the catalytic site are shown in detail: Asp5, Thr6, Glu41, Asp97 and Glu118 (residues position in the target - Fig 5C). Additionally, superimposition showed the matching of the Cys9, involved in the VapC dimerization, positioned in the neighborhood of the catalytic site, which might indicate the participation of the cysteine in the catalytic mechanism of the cleavage of tRNA^fMet^ by VapC.

The analysis of the quality scores of the 3D models constructed for leptospiral VapC showed that *Shigella*'s VapC is the better template in comparison with the other VapC experimentally solved structures, as denoted by the evaluation scores (Table 2). Furthermore, despite the higher sequence identity between *Salmonella*'s and *Shigella*'s VapCs (79%) than that between *Leptospira*'s and S*higella*'s VapCs (38%), the scores of leptospiral VapC model are slightly higher than the scores of *Salmonella*'*s* VapC model, based on *Shigella*'s VapC template (Table 2). This result indicates that sequence similarity, although important, is not decisive for this evaluation.

**Table 2:** Evaluation of VapC models.

Target VapC structure	Template VapC structure – pdb	Qmean4	Z score
*L. interrogans* (*)	*S. flexneri* – 3TND/C	0.769	−0.34
*L. interrogans*	*N. gonorrhoeae* (FitAB) – 2H1C/A	0.533	−1.89
*L. interrogans*	*M. tuberculosis* (VapC5) – 3DBO	0.382	−2.93
*L. interrogans*	*M. tuberculosis* (VapC3) – 3H87/B	0.290	−5.41
*L. interrogans*	*P. aerophilum* (PAE0151) – 2FE1/A	0.180	−3.82
*S. enterica* (**)	*S. flexneri* – 3TND/C	0.753	−0.49
*M. tuberculosis* (VapC20) (^#^)	*S. flexneri* – 3TND/C	0.343	−4.83

VapC structural models were evaluated by QMEAN4 and Z score in order to compare and rank alternative models of the same target. QMAN4 is a reliability score consisting of a combination of four structural descriptors which ranges between 0 and 1 with higher values for better models. Z-score provides an estimate of the “degree of nativeness” of the structural features observed in a model; ‘good-quality’ models reach a mean *Z*-score of −0.65, ‘medium-quality’ -1.75, and the ‘low-quality’ −3.85. The analysis of the model quality scores showed that *Shigella* VapC is the only template to render a “good” model for VapC from *Leptospira* (*), as “good” as the one created for *Salmonella* VapC (**), which shares 89% identity with the temp**l**ate. In opposition, *Mycobacterium* VapC20 model (^#^) displayed low scores.

### VapC cleaves the initiator tRNA^fMet^

**Figure 6:**
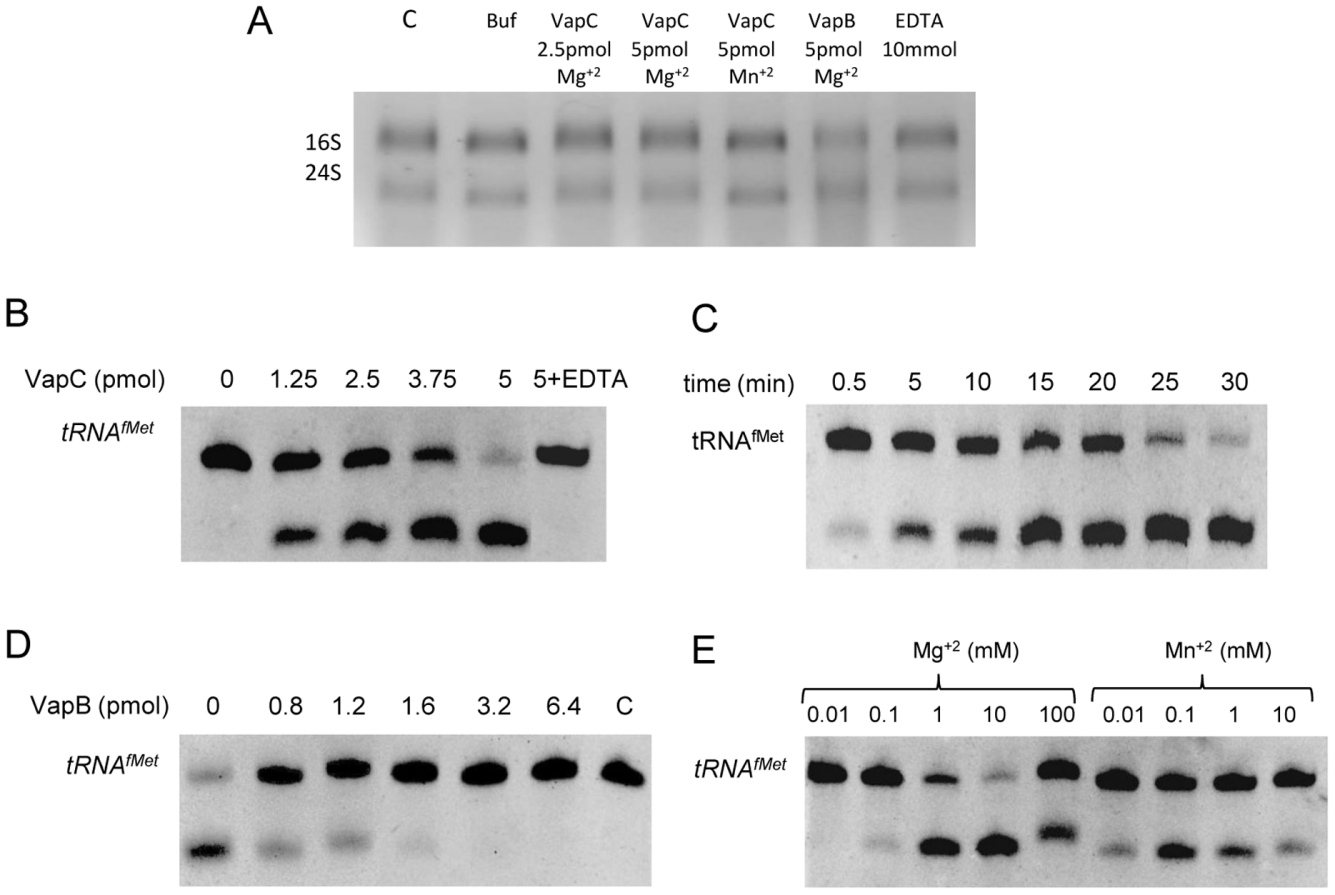
Leptospiral VapC toxin cleaves tRNA^fMet^ and is inhibited by the VapB antitoxin. Unless specified, the reactions were carried out under the following general condition: 5 pmol VapC was incubated with 3 ρmol of tRNA^fMet^ in 10 mM Hepes pH 7.5, 15 mM KCl, 1 mM DTT, 10 mM MgCl and 10% glycerol at 37°C for 30 min and analyzed by denaturing 8% PAGE 6 M urea, stained with ethidium bromide. Digestion of tRNA^fMet^ resulted in only one band indicating that it contains two fragments of same size. (**A**) VapC showed no activity over *E. coli* rRNA. VapC (2.5 and 5 pmol) was incubated with 1 µg of rRNA at 37°C for 10 min and analyzed by 1% Tris-Acetic-EDTA ethidium bromide agarose gel electrophoresis. (**B**) VapC activity is dose dependent. The initiator tRNA^fMet^ was incubated with increasing amounts of VapC (0 to 5 pmol). Incubation with 10 mM EDTA abrogates RNAse activity. (**C**) Rnase activity is time dependent. VapC was incubated with the substrate for 0.5 to 30 min. (**D**) VapC activity is inhibited by VapB. VapC was pre-incubated for 15 min with increasing amounts of VapB (0 to 6.8 pmol), before the addition of tRNA^fMet^. (**E**) VapC cleaves tRNA^fMet^ more effectively using Mg^+2^ than Mn^+2^. VapC aliquots were incubated with Mg^+2^ (0.01 to 100 mM) or Mn^+2^ (0.01 to 10 mM). Each assay was performed at least twice.

VapCs from *H. influenzae* [8], *M. tuberculosis* [22], [23] and *P. salmonis* [24], among others, have been confirmed to present the ribonuclease activity, ascribed to PIN domains, towards *E. coli* total RNA (mainly rRNA), mRNA or synthetic substrates. Likewise recently described for VapCs from *S. flexneri* and *S. enterica* [25], we did not observe any activity of the leptospiral VapC over *E. coli* total RNA (Fig. 6A) or DNA (data not shown). Furthermore, considering that VapC from *S. flexneri* was found to cleave tRNA^fMet^ [25], and that its X-ray structure matched closely with the 3D model of VapC from *L. interrrogans*, we decided to test the activity of leptospiral VapC towards the initiator tRNA^fMet^.

In our experiments, leptospiral VapC was shown to cleave *E. coli* tRNA^fMet^ in a dose and time dependent manner (Fig. 6B and 6C) and to be inhibited by VapB (Fig. 6D), also in a dose dependent manner. We confirmed that VapC ribonuclease activity is dependent on divalent metal ions, being abolished by the addition of EDTA (Fig. 6B). The effect of Mg^+2^ and Mn^+2^ ions were compared (Fig. 6E) and revealed that VapC achieves a higher rate of tRNA^fMet^ consumption with Mg^+2^ than with Mn^+2^, and that they act at different optimal concentrations: 10 mM and 0.1 mM, respectively. The substrate tRNA^fMet^ is a highly conserved molecule, being identical in *L. interrogans*, *E. coli* and *S. flexneri*. Although we have not determined the exact site of the leptospiral VapC cleavage, the digestion of RNA^fMet^ resulted in only one band, suggesting that the substrate might be cleaved at the same point reported to the enteric VapCs (between nucleotides 38 and 39) [25], which results in two fragments of the same size (38 nucleotides).

## Discussion

The understanding of the essential role of Toxin-Antitoxin systems, suggested by their ubiquitous distribution among bacteria and their success through evolution, still represents a challenge for scientists. Indeed, their precise functions and mechanisms of action are far from being fully elucidated, in part due to the wide range of possibilities opened up by their different types and different families that diverge substantially in sequences, targets and functions.

Concurring to the task of finding out a more comprehensive explanation for the role of this intriguing system, this work provides a new method consisting of long term expression and the use of HPP to obtain soluble active VapC, and adds information on the function of the leptospiral VapBC module.

The pull down and ligand affinity blotting experiments showed that the recombinant leptospiral VapC toxin and its cognate VapB antitoxin interact with each other, thereby resulting in the neutralization of the toxic effects of VapC. This interaction probably represents the ultimate mechanism by which the activity of the toxin is regulated in the native bacterium.

We showed that VapC from *L. interrogans* cleaves tRNA^fMet^, sharing the same biological target with the enteric VapCs. It is important to mention that, despite of the relative large number of publications on this field, it is the first time that a coincidental function is attributed to VapCs from unrelated organisms. This results open up new perspectives on the use of comparative structural studies to identify the features that define substrate specificities within these family members.

Several studies have shown the ability of VapCs to act on bacterial ribosomal RNA [8], [22], [24]. The degradation pattern of the RNA observed in these works suggests that the referred toxins act as nonspecific ribonucleases. On the other hand, it was reported a methodology to determine the precise RNA cutting sites of different VapCs by combining the use of RNA pentaprobes (RNA transcribed from a set of plasmids whose overlapping inserts encode every combination of bases) as substrate and analysis of the products by MALDI-TOF MS [38]. The study showed that VapCs recognize not only a specific RNA sequence, but also that the RNA secondary structure is important as a determinant of enzyme specificity, adding complexity to the establishment of VapC cellular targets. Although revealing specific cutting sites only in an artificial substrate, the authors used this method to find possible biological targets in *M. tuberculosis* by searching the revealed sequence tags on specific mRNAs, which decaying was observed by microarray assay during TA induction [27].

Going further on the search for biological substrates, recent reports identified the target of VapC from the enteric bacteria, which cleave solely the initiator tRNA^fMet^ [26], and the target of VapC20 from *M. tuberculosis*, which cleaves specifically the Sarcin-Rich loop (SRL) of the 23S ribosomal RNA [26]. Through mutational analysis of SRL, it was shown that changes in the primary sequence neighboring of the reactive site are less fundamental to activity than the conformation of the substrate and also that the full RNA molecule, but not only SRL, is necessary for cleavage. This analysis shed light on the fact that structures far from the vicinity of the reactive site of the substrate and/or ribosomal proteins may interact with VapC, therefore playing a pivotal role in cleavage mechanism. According to the authors, subtle structural differences could explain why VapC from *S. flexneri* and VapC20 from *M. tuberculosis*, which are most likely to be structurally very similar, would cleave distinct RNA targets [26].

Indeed, it is likely that the high divergence on the amino acid sequences within VapC family members would lead to structural changes which would somehow be responsible for the substrate specificities. Interestingly, the 3D model of VapC20, which cleaves SRL, created based on the experimental X-ray structure of VapC from *S. flexneri*, which cleaves tRNA^fMet^, presented bad reliability scores (Table 02). In opposition, the 3D model created for leptospiral VapC (Fig. 5A), also based on the template of *Shigella*'s VapC displayed excellent reliability scores (Table 02), indicating that both proteins, which share the same target, are structurally close related. These data indicate that our approach, based on the evaluation scores of structural models, may contribute, even in the absence of experimental structural data, to an initial attempt of relating VapCs 3D structure to their biological targets. The elucidation of the experimental 3D structure of leptospiral VapC is necessary to confirm this approach, objective for which we are currently working.

To date, this is the first report that describes the ribonuclease activity of the leptospiral VapC and the second to show a VapC toxin cleaving the initiator tRNA^fMet^, suggesting that it might be a mechanism of action common to a larger group of bacteria. As demonstrated for enteric VapCs, the cleavage of the initiator tRNA should affect translation in a global manner in leptospira. This study contributes to elucidating the functions of TA modules, in particular of the VapBC module of *Leptospira interrogans*, reinforcing the role of TA in post-transcriptional regulation. Furthermore, considering the significant differences between the amino acid sequences of *Leptospira* and *Shigella*'s VapCs in opposition to their structural matching and same specific target, one can speculate on the major role of the evolutionary selection pressure over the 3D structure than over the primary structure of these proteins.
